# YejM Modulates Activity of the YciM/FtsH Protease Complex To Prevent Lethal Accumulation of Lipopolysaccharide

**DOI:** 10.1128/mBio.00598-20

**Published:** 2020-04-14

**Authors:** Randi L. Guest, Daniel Samé Guerra, Maria Wissler, Jacqueline Grimm, Thomas J. Silhavy

**Affiliations:** a Department of Molecular Biology, Princeton University, Lewis Thomas Laboratory, Princeton, New Jersey, USA; National Cancer Institute

**Keywords:** lipopolysaccharide, proteolysis, LpxC, FtsH, YciM, YejM, outer membrane

## Abstract

Lipopolysaccharide (LPS) is an essential glycolipid present in the outer membrane (OM) of many Gram-negative bacteria. Balanced biosynthesis of LPS is critical for cell viability; too little LPS weakens the OM, while too much LPS is lethal. In Escherichia coli, this balance is maintained by the YciM/FtsH protease complex, which adjusts LPS levels by degrading the LPS biosynthesis enzyme LpxC. Here, we provide evidence that activity of the YciM/FtsH protease complex is inhibited by the essential protein YejM. Using strains in which LpxC activity is reduced, we show that *yciM* is epistatic to *yejM*, demonstrating that YejM acts upstream of YciM to prevent toxic overproduction of LPS. Previous studies have shown that this toxicity can be suppressed by deleting *lpp*, which codes for a highly abundant OM lipoprotein. It was assumed that deletion of *lpp* restores lipid balance by increasing the number of acyl chains available for glycerophospholipid biosynthesis. We show that this is not the case. Rather, our data suggest that preventing attachment of *lpp* to the peptidoglycan sacculus allows excess LPS to be shed in vesicles. We propose that this loss of OM material allows continued transport of LPS to the OM, thus preventing lethal accumulation of LPS within the inner membrane. Overall, our data justify the commitment of three essential inner membrane proteins to avoid toxic over- or underproduction of LPS.

## Abstract (precis)

Gram-negative bacteria are encapsulated by an outer membrane (OM) that is impermeable to large and hydrophobic molecules. As such, these bacteria are intrinsically resistant to several clinically relevant antibiotics. To better understand how the OM is established or maintained, we sought to clarify the function of the essential protein YejM in Escherichia coli. Here, we show that YejM inhibits activity of the YciM/FtsH protease complex, which regulates synthesis of the essential OM glycolipid lipopolysaccharide (LPS). Our data suggest that disrupting proper communication between LPS synthesis and transport to the OM leads to accumulation of LPS within the inner membrane (IM). The lethality associated with this event can be suppressed by increasing OM vesiculation. Our research has identified a completely novel signaling pathway that we propose coordinates LPS synthesis and transport.

## INTRODUCTION

Nearly all bacteria are surrounded by a multilayered envelope that separates the cytoplasm from the external environment (1). In both Gram-positive and Gram-negative bacteria, this envelope consists of a cytoplasmic/inner membrane (IM) and the peptidoglycan sacculus. Gram-negative bacteria contain an additional layer called the outer membrane (OM), which lies on the external face of the peptidoglycan. The OM is an asymmetric lipid bilayer with the glycolipid lipopolysaccharide (LPS) in the outer leaflet and glycerophospholipids (PLs) in the inner leaflet (2). This membrane forms a robust permeability barrier that substantially slows the influx of large and hydrophobic molecules and works together with the peptidoglycan to provide mechanical strength to the cell (3). Both functions arise from the strong lateral interactions between neighboring molecules of LPS, which are densely packed within the outer leaflet.

The basic structure of LPS consists of the glucosamine-based phospholipid lipid A connected to a core oligosaccharide (lipid A-core) (4). Additional polysaccharides can be attached to lipid A-core, including O antigen, enterobacterial common antigen, and colanic acid (5–7). Notably, laboratory strains of Escherichia coli K-12 do not synthesize O antigen, and modification of LPS with enterobacterial common antigen or colonic acid is rare under normal growth conditions (6–8). As such, the majority of LPS in E. coli K-12 is in the form of lipid A-core. Synthesis of lipid A-core occurs on the cytoplasmic face of the IM (4). In E. coli, the first step in lipid A biosynthesis is catalyzed by LpxA, which attaches a single acyl chain to UDP-GlcNAc (UDP-*N*-acetylglucosamine) to form UDP-monoacyl-GlcNAc (9). UDP-monoacyl-GlcNAc is then deacetylated by LpxC (10), which makes room for a second acyl chain to be added. The reaction catalyzed by LpxA has an unfavorable equilibrium constant, and as such, LpxC performs the first committed step of lipid A biosynthesis (10, 11). The amount of LpxC is regulated by the protease FtsH (12). Delivery of LpxC to FtsH is mediated by the adaptor protein YciM (13), an integral IM protein with a cytoplasmic region containing nine tetratricopeptide repeat (TPR) motifs and a rubredoxin-like domain (14–16). Null mutations in either *ftsH* or *yciM* are lethal due to increased LPS biosynthesis (13, 16), which is thought to deplete the cell of acyl chains that are needed for PL biosynthesis (12). Mutations that rebalance the ratio of LPS to PLs by either lowering LPS or increasing PL biosynthesis suppress deletion of *ftsH* or *yciM* (12, 13). It has previously been shown that deletion of the highly abundant lipoprotein Lpp, which tethers the OM to the underlying peptidoglycan, can suppress deletion of *yciM* (13, 16–18). As Lpp is anchored to the OM by three acyl chains (19), it has been hypothesized that loss of *lpp* restores lipid balance in the Δ*yciM* mutant by increasing the number of acyl chains available for PL biosynthesis (16, 20).

YejM is an essential IM protein containing a nonessential C-terminal globular domain that extends into the periplasmic space between the inner and outer membranes (21–23). The membrane and globular domains are connected by a basic linker region that is required for YejM function but is not essential for viability. E. coli lacking the globular domain and linker region of YejM displays several phenotypes that are characteristic of OM barrier defects, including sensitivity to large and hydrophobic antibiotics, impaired growth at elevated temperatures, and leakage of periplasmic proteins (24, 25). It has also been reported that *yejM* mutants have a lower ratio of LPS to PLs, suggesting that loss of YejM activity may alter OM lipid synthesis (24, 25). More recent studies suggest that YejM transports cardiolipin to the OM (23). However, this does not explain why *yejM* is essential, since E. coli can survive without cardiolipin (26). Moreover, the OM defects caused by truncation of YejM persist in the absence of cardiolipin (27). As such, the essential function performed by YejM that impacts OM integrity remains completely unknown.

In this study, we show that YejM acts upstream of YciM to restrain degradation of LpxC by FtsH. We also show that simply preventing attachment of Lpp to the peptidoglycan by a mutation that does not impact protein expression or acylation prevents the lethality caused by unrestrained synthesis of LPS. We propose that this lethality occurs because of LPS in the IM. Loss of OM material due to hypervesiculation in the *lpp* mutants allows LPS transport to keep up with this unrestrained synthesis, preventing toxic accumulation within the IM. Overall, our data suggest that the essential function of YejM is to control activity of the YciM/FtsH protease complex.

## RESULTS

### Mutations in *yciM* and *lpxC* suppress the OM defect of the *yejM*569 mutant.

**FIG 1:**
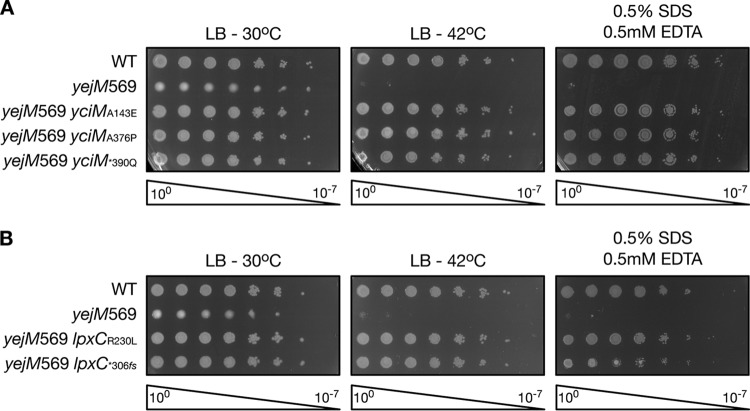
Mutations in *yciM* and *lpxC* suppress the outer membrane (OM) defect of the *yejM*569 mutant. Serial dilutions of the indicated strains were spotted onto LB or LB supplemented with 0.5% SDS and 0.5 mM EDTA. Bacteria spotted on LB were grown at 30°C or 42°C while those spotted on LB containing SDS and EDTA were grown at 30°C. (A) *yciM*_A143E_, *yciM*_A376P_, and yciM_*390Q_ rescue growth of the *yejM*569::*cam* mutant under nonpermissive conditions. All strains contain the *ycjM*::Tn*10* allele, which is genetically linked to *yciM*. The strains shown are RLG431, RLG467, RLG507, RLG509, and RLG511. (B) *lpxC*_R230L_ and lpxC_*390_*_fs_* allow E. coli containing the *yejM*569::*cam* allele to grow under nonpermissive conditions. All strains contain the *leuB*::Tn*10* allele, which is linked to *lpxC*. The strains shown are RLG433, RLG547, RLG548, and RLG550. Data are representative of three independent experiments. WT, wild type; *, stop codon; *fs*, frameshift.

To clarify the function of YejM, we screened for suppressors of the OM permeability and temperature sensitivity phenotypes of E. coli expressing *yejM*569, a truncated version of YejM lacking its globular and linker domains. It has previously been shown that the temperature sensitivity of E. coli expressing the truncated YejM can be restored by expressing a wild-type copy of *yejM* in *trans*, suggesting that removing the globular and linker domains reduces YejM activity (21). Cells expressing *yejM*569 were incubated under nonpermissive conditions (42°C or SDS/EDTA) until suppressor colonies grew. We identified several mutations in *yciM* and *lpxC* that suppress both the OM permeability defect and the temperature sensitivity of *yejM*569 (Fig. 1). One suppressor in YciM exchanged an alanine for glutamic acid at residue 143 in TPR four, while another contains an alanine-to-proline substitution at reside 376 in the rubredoxin-like domain. Mutation of the stop codon in YciM to glutamine, which extends the protein by eight amino acids, also suppresses the OM defect of the *yejM*569 mutant (Fig. 1A). One mutation in LpxC that suppress *yejM*569 exchanges a conserved arginine for a leucine at residue 230. LpxC*^306^*^fs^* contains a frameshift mutation that removes two nucleotides in the stop codon and extends the protein by 20 amino acids. Given that both YciM and LpxC are involved in lipid A biosynthesis (10, 13), our data suggest that the OM defect caused by loss of the periplasmic domain of YejM can be corrected by modulating the level of LPS.

### LPS levels are reduced in the truncated *yejM* mutant.

**FIG 2:**
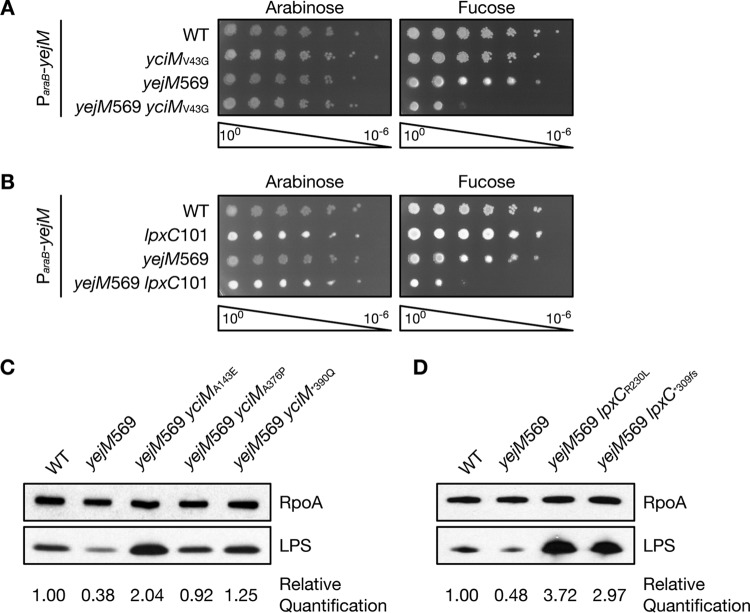
*yejM*569 lowers lipopolysaccharide (LPS) levels and mutations that suppress *yejM*569 increase LPS. (A and B) Tenfold serial dilutions of the indicated strains containing a plasmid expressing wild-type (WT) *yejM* from an arabinose-inducible promoter were grown on LB containing 0.2% arabinose or 0.05% fucose at 30°C. (A) The *yciM*_V43G_ *yejM*569 double mutant fails to grow unless a wild-type copy of *yejM* is expressed. All strains contain the *ycjM*::Tn*10* marker. (B) The *lpxC*101 *yejM*569 double mutant cannot grow unless wild-type *yejM* is expressed. (C and D) LPS levels were determined by immunoblot analysis using antibodies that recognize LPS. Antibodies recognizing RpoA were used as a loading control. LPS levels in the *yejM*569 mutant are decreased. Relative quantification indicates LPS levels compared to the levels in the wild type. (C) Suppressor mutations in *yciM* increase LPS levels in the *yejM*569 mutant. All strains contain *ycjM*::Tn*10*. (D) Suppressor mutations in *lpxC* increase LPS in the *yejM*569 mutant. All strains contain the *leuB*::Tn*10* allele. Data are representative of three independent experiments. *, stop codon; *fs*, frameshift.

While our data suggest that altering LPS biosynthesis suppresses the OM defect of the *yejM*569 mutant, it was not clear whether the suppressors increase or decrease LPS levels. To investigate this, we determined whether a gain-of-function mutation in *yciM* (*yciM*_V43G_) that decreases levels of both LpxC and LPS (28, 29) can suppress *yejM*569. We were unable to introduce the *yejM*569 allele into E. coli expressing *yciM*_V43G_, suggesting that these mutations may be synthetically lethal. To test this hypothesis, we introduced *yejM*569 into a *yciM*_V43G_ mutant expressing a wild-type copy of *yejM* from an arabinose-inducible promoter. In the presence of arabinose, the *yejM*569 *yciM*_V43G_ double mutant is viable (Fig. 2A). However, when expression of wild-type *yejM* is repressed by adding fucose to the growth medium, the *yejM*569 *yciM*_V43G_ double mutant fails to grow (Fig. 2A). Depletion of *yejM* with fucose did not impair growth of the *yciM*_V43G_ single mutant, which encodes a wild-type copy of *yejM* at the native locus, or the *yejM*569 single mutant (Fig. 2A). These results demonstrate that *yejM*569 and *yciM*_V43G_ are synthetically lethal and suggest that lowering LPS levels in the *yejM*569 mutant is toxic.

To confirm that decreased LPS levels prevent growth of the *yejM*569 mutant, we determined whether *yejM*569 is synthetically lethal with *lpxC*101, an allele of *lpxC* that decreases lipid A biosynthesis (10, 30, 31). *yejM*569 was introduced into a *lpxC*101 mutant expressing a wild-type copy of *yejM* from an arabinose-inducible promoter, and the ability of this strain to grow in the presence of arabinose or fucose was assessed. Much like the *yciM*_V43G_ mutant, the *yejM*569 *lpxC*101 double mutant could not grow when *yejM*569 was the sole copy expressed (Fig. 2B). These data provide additional evidence to suggest that the *yejM*569 mutant fails to grow when LPS levels are decreased. As such, it is likely that mutations in *yciM* and *lpxC* that suppress the OM defect caused by truncation of YejM act to increase LPS.

Our data suggest that the *yejM*569 mutation alone may lower LPS levels, and when paired with additional mutations that also lower LPS, the combined decrease cannot support growth. To quantify levels of LPS, we utilized immunoblot analysis with an antibody raised against LPS. We observed an approximate twofold decrease in LPS levels in the *yejM*569 mutant compared to that of the wild type (Fig. 2C and D). Mutations in *yciM* and *lpxC* that suppress the OM defect of the *yejM*569 mutant increase LPS levels in this background to different extents. LPS levels in the *yejM*569 *yciM*_A376P_ double mutant were higher than that of the *yejM*569 single mutant but lower than that of the wild type (Fig. 2C). LPS levels in all other suppressors were higher than in both the *yejM*569 single mutant and wild type. Compared to the wild type, LPS levels in the *yejM*569 mutants expressing *yciM*_A143E_, yciM_*390Q_, and lpxC_*309_*_fs_* were increased by 2.04-, 1.25-, and 2.97-fold, respectively (Fig. 2C and D). The *yejM*569 *lpxC*_R230L_ mutant had the highest level of LPS, which was nearly fourfold higher than that of the wild type (Fig. 2D). Taken together, these data suggest that loss of the periplasmic domain in YejM lowers LPS levels and that the OM defect in this mutant can be corrected by increasing LPS.

### The LpxC degradation pathway is activated in the *yejM*569 mutant.

**FIG 3:**
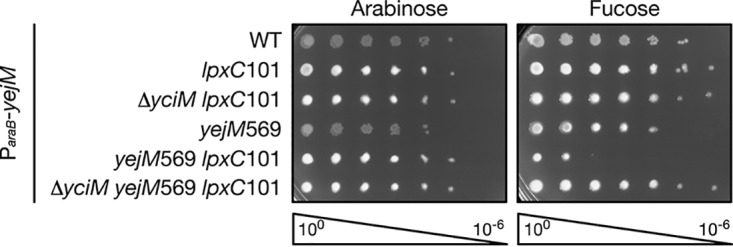
*yciM* is epistatic to *yejM*569. Serially diluted strains were plated on LB supplemented with 0.2% arabinose or 0.05% fucose. All strains contain the *ycjM*::Tn*10* marker and a plasmid encoding a wild-type copy of *yejM* from an arabinose-inducible promoter. Deletion of *yciM* rescues the synthetic lethality of the *yejM*569 *lpxC*101 double mutant, suggesting that *yejM* functions upstream of *yciM* to regulate LPS. Data are representative of three biological replicates.

We next wanted to determine how *yejM*569 lowers LPS levels. Clues as to how this may occur come from the mutations that suppress the OM defect of the *yejM*569 mutant. *yciM*_A143E_, *yciM*_A376P_, and yciM_*390Q_ increase LPS levels in the *yejM*569 strain, suggesting that these mutations likely stabilize LpxC by impairing activity of YciM. Furthermore, the lpxC_*306_*_fs_* mutation extends the carboxy terminus of LpxC, which has previously been shown to block degradation by FtsH (32). As such, *yejM*569 may reduce LPS levels by increasing degradation of LpxC by the YciM/FtsH protease complex. To test this hypothesis, we took advantage of the synthetic lethality between *yejM*569 and *lpxC*101. If the *yejM*569 *lpxC*101 double mutant fails to grow because degradation of LpxC101 is increased, then preventing LpxC101 degradation by deleting *yciM* would restore viability. Alternatively, if *yejM*569 lowers LPS independently of the YciM/FtsH protease complex, then the *yejM*569 *lpxC*101 double mutant would fail to grow even in the absence of *yciM*. *yciM* was deleted in the *yejM*569 *lpxC*101 double mutant expressing a wild-type copy of *yejM* from an arabinose-inducible promoter. Notably, *yciM* is not essential in the *lpxC*101 background as LPS levels are decreased (13) (Fig. 3). As we observed previously (Fig. 2B), the *yejM*569 *lpxC*101 double mutant does not grow when wild-type *yejM* is depleted (Fig. 3). However, the *yejM*569 *lpxC*101 Δ*yciM* triple mutant is viable when only *yejM*569 is expressed (Fig. 3). These data are consistent with the hypothesis that *yejM*569 lowers LPS levels by increasing activity of the YciM/FtsH protease complex.

### YejM is not essential when the YciM/FtsH protease complex is inactivated.

**FIG 4:**
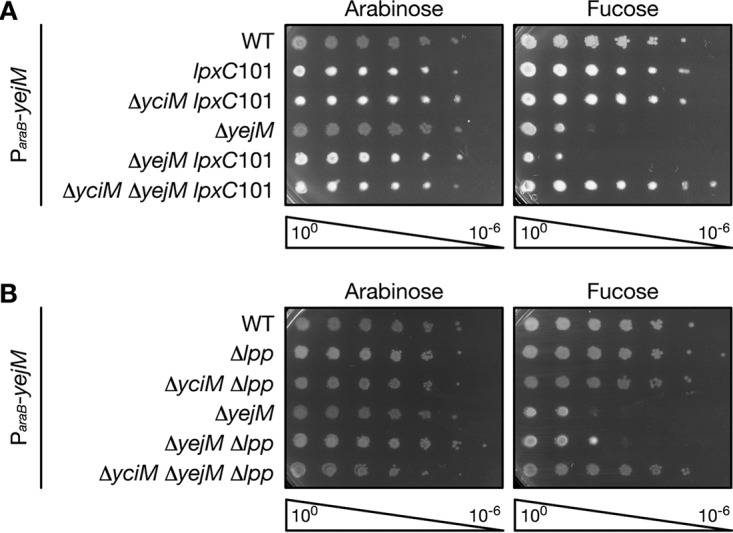
*yejM* is not essential in E. coli lacking *yciM*. Wild-type E. coli or E. coli containing the indicated mutations were serially diluted and plated on LB containing 0.2% arabinose or 0.05% fucose. All strains express a wild-type copy of *yejM* from an arabinose-inducible promoter on a plasmid. Deletion of *yciM* was suppressed with *lpxC*101 (A) or by deleting *lpp* (B). Data are representative of three biological replicates.

Our data suggest that truncation of YejM stimulates degradation of LpxC by the YciM/FtsH protease complex. As such, we hypothesized that loss of the entire YejM protein is lethal because the YciM/FtsH protease complex is hyperactivated. To test this hypothesis, we determined whether *yejM* is essential in E. coli lacking *yciM*. As *yciM* is also essential, we suppressed deletion of *yciM* by lowering the level of LPS with the *lpxC*101 mutation. The chromosomal copy of *yejM* was deleted in wild-type E. coli, as well as the *lpxC*101 single mutant and the Δ*yciM lpxC*101 double mutant each expressing a wild-type copy of *yejM* from an arabinose-inducible promoter. Growth of the YejM depletion strain is arabinose dependent (Fig. 4A), confirming that *yejM* is essential. The *lpxC*101 mutation did not rescue growth of the YejM depletion strain on fucose (Fig. 4A), demonstrating that lowering LPS levels does not suppress deletion of *yejM*. However, depletion of YejM is tolerated in the Δ*yciM lpxC*101 double mutant (Fig. 4A), suggesting that the *yejM* is not essential in E. coli lacking *yciM*.

To ensure that suppressing depletion of YejM is due to deletion of *yciM* and is not specific to the Δ*yciM lpxC*101 double mutant, we examined whether YejM can be depleted in a Δ*yciM* mutant that has been suppressed through a different mechanism. Previous studies have shown that deletion of *yciM* is tolerated in E. coli lacking *lpp* (13, 16). It is thought that deletion of *lpp* restores OM lipid balance in the *yciM* mutant by increasing the number of acyl chains available for PL biosynthesis (16, 20). As seen in Fig. 4B, deletion of *lpp* does not suppress depletion of YejM. However, YejM can be depleted in the Δ*yciM* Δ*lpp* double mutant (Fig. 4B), confirming that *yejM* is not essential when *yciM* is absent. Together, these results demonstrate that the essential function performed by YejM is dependent on YciM and suggest that loss of *yejM* may hyperactivate the YciM/FtsH protease complex.

### Mutations in *lpp* suppress loss of YciM by lowering LPS.

**FIG 5:**
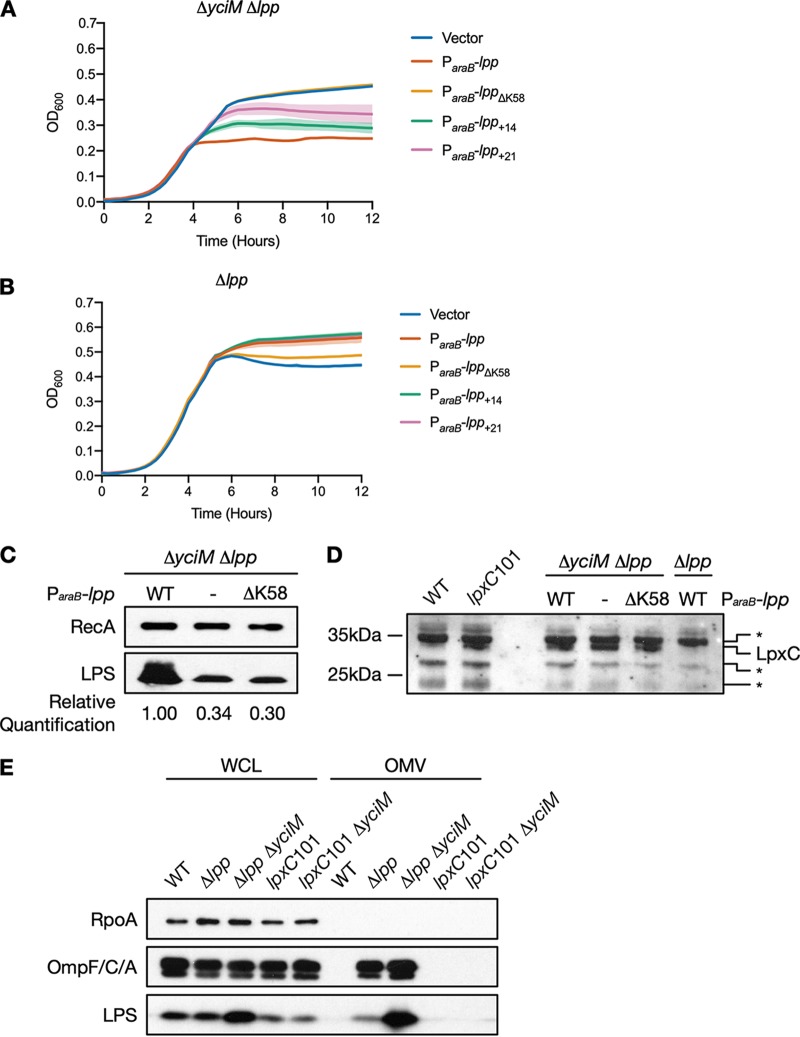
Mutations in *lpp* suppress deletion of *yciM* by decreasing levels of LPS. (A and B) Growth of the Δ*yciM* Δ*lpp* double mutant (A) and the Δ*lpp* single mutant harboring plasmid pBAD18 or pBAD18 expressing wild-type *lpp*, *lpp*_ΔK58_, *lpp*_+14_, or *lpp*_+21_ from an arabinose-inducible promoter (B). Bacteria were diluted into LB supplemented with 0.5% arabinose. OD_600_, which was used as a proxy for growth, was measured every 15 min for 12 h. Data represent the means and standard deviations of three biological replicates. (C) Immunoblot analysis of LPS levels in the Δ*yciM* Δ*lpp* double mutant containing pBAD18 or pBAD18 expressing wild-type *lpp* or *lpp*_ΔK58_ from an arabinose-inducible promoter. RecA was used as a loading control. (D) Immunoblot analysis of LpxC levels in the Δ*yciM* Δ*lpp* double mutant containing pBAD18 or pBAD18 expressing wild-type *lpp* or *lpp*_ΔK58_ from an arabinose-inducible promoter. As LpxC levels are increased in the Δ*yciM* mutant (13, 16), the band corresponding to LpxC was identified by comparing Δ*yciM* Δ*lpp* double mutants and a Δ*lpp* single mutant expressing wild-type *lpp* from an arabinose-inducible promoter. This band was confirmed to be LpxC using the *lpxC*101 mutation, which significantly increases LpxC levels (29). The presence of a nonspecific band is indicated by an asterisk. (E) OM vesicles in the wild type, the Δ*lpp* and *lpxC*101 single mutants, and the Δ*lpp* Δ*yciM* and *lpxC*101 Δ*yciM* double mutants. RpoA was used as a cytoplasmic marker, while OmpC/F/A and LPS were used as markers of the OM. Increased amounts of OmpC/F/A and LPS in the OM vesicle fraction indicate that the Δ*lpp* and Δ*lpp* Δ*yciM* mutants hypervesiculate. Data in panels C to E are representative of two or more independent experiments. Lanes in panels C to E: −, vector; WT, wild type; WCL, whole-cell lysate; OMV, outer membrane vesicles.

Previous studies have proposed that elevated LPS biosynthesis resulting from deletion of *ftsH* or *yciM* is lethal due to reduced availability of acyl chains required for PL biosynthesis (12, 16, 33). To investigate whether deletion of *lpp* suppresses deletion of *yciM* by liberating acyl chains normally sequestered by this abundant lipoprotein (16, 20), we determined whether deletion of *yciM* is tolerated in E. coli lacking the C-terminal lysine residue that attaches Lpp to the peptidoglycan sacculus (34). Unhooking Lpp from the peptidoglycan leads to many of the same phenotypes as deleting *lpp* entirely (17). However, mutating the K58 residue does not affect Lpp expression or localization to the OM (35), suggesting that Lpp^ΔK58^ is lipidated and would sequester acyl chains like the wild type. We compared growth of a Δ*yciM* Δ*lpp* double mutant containing a plasmid expressing wild-type *lpp* or *lpp*_ΔK58_ from an arabinose-inducible promoter to that of a Δ*yciM* Δ*lpp* mutant containing an empty vector. When grown in the presence of arabinose, the Δ*yciM* mutant that does not carry *lpp* grew better than the Δ*yciM* mutant expressing wild-type *lpp* (Fig. 5A), confirming that loss of *lpp* suppresses deletion of *yciM*. The period of exponential growth in the Δ*yciM* mutant expressing wild-type *lpp* likely represents the time required to establish a strong covalent linkage between the OM and the peptidoglycan upon inducing *lpp* expression. Growth of the Δ*yciM* mutant expressing *lpp*_ΔK58_ was similar to that of the Δ*yciM* mutant that does not bear *lpp* (Fig. 5A), suggesting that loss of *lpp* does not suppress deletion of *yciM* by liberating acyl chains for lipid biosynthesis.

Unhooking Lpp from the peptidoglycan disrupts OM integrity, promotes OM vesiculation, and prevents proper communication across the periplasm by increasing the distance between the inner and outer membranes (17). To narrow down which of these phenotypes is responsible for suppressing deletion of *yciM*, we monitored growth of the Δ*yciM* Δ*lpp* mutant expressing a plasmid-borne, arabinose-inducible copy of *lpp*_+14_, a mutation that extends the length of Lpp by 14 amino acids (36). Both Lpp^ΔK58^ and Lpp^+14^ increase the length of the periplasm by an average of 3 nm; however, Lpp^+14^ does not affect OM permeability or vesiculation (36). The Δ*yciM* mutant expressing *lpp*_+14_ grows better than that expressing wild-type *lpp*, but worse than the Δ*yciM* mutant lacking *lpp* entirely (Fig. 5A). Further increasing the distance between the inner and outer membranes by extending the length of Lpp by 21 amino acids (36, 37) leads to better growth than when *lpp*_+14_ is expressed (Fig. 5A). However, the Δ*yciM* mutant expressing *lpp*_+21_ still does not grow as well as the Δ*yciM* mutant lacking *lpp* (Fig. 5A). Expression of *lpp*_+14_ and *lpp*_+21_ is not toxic in cells expressing a wild-type copy of *yciM* (Fig. 5B), indicating that impaired growth of the Δ*yciM* mutants expressing *lpp*_+14_ and *lpp*_+21_ is specifically due to the loss of *yciM*. Given that *lpp*_+14_ and *lpp*_+21_ do not suppress deletion of *yciM* to the same extent as deletion of *lpp* or *lpp*_ΔK58_, it is unlikely that loss of *lpp* suppresses deletion of *yciM* by increasing the distance between the inner and outer membrane.

As many of the mutations that suppress deletion of *yciM* are known to affect LPS levels (13, 16), we hypothesized that mutating *lpp* may alter LPS levels in E. coli lacking *yciM*. In support of this hypothesis, deletion of *lpp* has been shown to suppress the lethality associated with hyperactivation of the OM phospholipase PldA by decreasing LPS levels (28). We found that the Δ*yciM* mutants that lacked *lpp* or expressed *lpp*_ΔK58_ had approximately 2.5-fold-less LPS than the Δ*yciM* mutant expressing wild-type *lpp* (Fig. 5C). These data suggest that loss of Lpp function suppresses deletion of *yciM* by decreasing levels of LPS. To determine whether loss of Lpp in the Δ*yciM* mutant affects LPS biosynthesis, we measured levels of LpxC. We found that the level of LpxC in the Δ*yciM* mutant lacking *lpp* or expressing *lpp*_ΔK58_ is similar to that expressing wild-type *lpp* (Fig. 5D), suggesting that loss of Lpp or unhooking Lpp from the peptidoglycan may reduce LPS levels without affecting LPS biosynthesis. Instead, it is likely that loss of Lpp function lowers LPS by increasing OM vesiculation. To test this hypothesis, we quantified OM vesicles in wild-type E. coli, the Δ*lpp* single mutant, and the Δ*lpp* Δ*yciM* double mutant. We found that OM vesicles are produced in both the Δ*lpp* and Δ*lpp* Δ*yciM* mutants and that OM vesicles from the Δ*lpp* Δ*yciM* mutant have increased LPS (Fig. 5E). OM vesicles are not detected in the Δ*yciM lpxC*101 mutant (Fig. 5E), suggesting that production of OM vesicles is specific to the Δ*lpp* Δ*yciM* double mutant.

## DISCUSSION

In this study, we sought to better understand the role of the essential protein YejM in OM biogenesis. Our data suggest that removing the nonessential globular and linker domains of YejM, which reduces YejM activity (21), lowers LPS levels in a *yciM*-dependent manner. Consequently, these cells are sensitive to detergents and cannot grow at elevated temperatures. As YciM is the adaptor protein that delivers LpxC to FtsH for degradation (13), it is likely that partial loss of YejM activity lowers LPS levels by increasing proteolysis of LpxC. These data suggest that complete loss of *yejM* is lethal because the YciM/FtsH protease complex is hyperactivated. Indeed, we found that *yejM* is no longer essential in E. coli lacking *yciM*. Overall, our findings indicate that YejM inhibits the YciM/FtsH protease complex, which is responsible for degrading LpxC. OM defects associated with truncation of YejM in Salmonella enterica serovar Typhimurium can also be suppressed by mutations in *yciM*, *lpxC*, and *ftsH* (38), suggesting that control of LpxC degradation may be a conserved function of YejM.

Several studies have shown that LpxC degradation is regulated by fatty acids. Lipid A and PLs use the same fatty acids, suggesting that competition must exist between these two biosynthetic pathways for precursors. As such, previous studies hypothesized that LpxC degradation is regulated to ensure that sufficient fatty acids are available for PL biosynthesis (12, 33). In support of this model, increased activity of the fatty acid biosynthesis enzyme FabZ stabilizes LpxC (12, 39). However, overexpression of other genes involved in fatty acid biosynthesis have the opposite effect on LpxC stability (39, 40). As such, it is unclear whether LpxC is regulated in order to balance biosynthesis of PLs and LPS (40). Aberrant accumulation of PLs in the outer leaflet of the OM, which denotes a greater need for LPS, also stabilizes LpxC (29). Fatty acids released upon breakdown of outer leaflet PLs by the phospholipase PldA are imported into the cytoplasm, where they inhibit activity of the YciM/FtsH protease complex. A recent study has shown that PldA is protective in E. coli lacking part of the YejM globular domain (27), suggesting that PldA activity may stabilize LpxC in the *yejM* mutant. LpxC stability is correlated with levels of the alarmone guanosine tetraphosphate (ppGpp) (41). LpxC is rapidly degraded during slow growth, when ppGpp levels are high. In contrast, during fast growth when ppGpp levels are low, LpxC is stabilized. This regulatory pattern is reversed when ppGpp is eliminated. Most recently, it was shown that LpxC is degraded upon overexpression of PyrH, an essential protein involved in pyrimidine biosynthesis (39). It is thought that PyrH overexpression may change levels of UDP-GlcNAc, a precursor for lipid A biosynthesis.

How these diverse signals are sensed by YciM and/or FtsH is not clear. Many of these signals affect production of lipid A disaccharide, an intermediate of lipid A biosynthesis (40). Computational modeling suggests that accumulation of lipid A disaccharide stimulates degradation of LpxC, a finding supported by experimental data (40). Whether YejM is required for lipid A disaccharide to regulate LpxC degradation is unknown. Previous studies have shown that the periplasmic domain of YejM is able to bind lipids (22, 23). However, it is unlikely that these regions of YejM are able to interact with lipid A disaccharide, which is located on the cytoplasmic side of the IM. Further studies are needed to test whether lipid A disaccharide is a key biosynthetic intermediate, and if so, how its levels are detected and integrated into the YejM and YciM/FtsH regulatory circuit.

Deleting *yciM* stabilizes LpxC, which leads to constitutive biosynthesis of LPS (13, 16). This lethal event can be suppressed by deleting *lpp*, which codes for a highly abundant lipoprotein that tethers the OM to the underlying peptidoglycan sacculus (13, 16–18). Here, we show that deletion of *yciM* is tolerated in E. coli producing a mutant version of Lpp (Lpp^ΔK58^) that cannot attach to the peptidoglycan (34). Given that Lpp^ΔK58^ is lipidated, these data challenge the notion that loss of Lpp suppresses deletion of *yciM* by increasing the number of acyl chains available for PL biosynthesis. Rather, our data suggest that suppression is due to greatly weakened anchoring of the OM to the peptidoglycan. It is well-known that Δ*lpp* and *lpp*_ΔK58_ mutants increase production of OM vesicles (36, 37, 42–44), and our data suggest that the Δ*yciM* Δ*lpp* double mutant produces OM vesicles. We propose that these vesicles help the cell to shed the excess LPS produced because of *yciM* deletion. In support of this hypothesis, OM vesicles produced in strains synthesizing larger amounts of LPS are enriched with lipids (44). Although we cannot exclude the possibility that deletion of *lpp* reduces LPS levels by affecting LPS biosynthesis at a step downstream of LpxC, we do not think this likely. A previous study has found that deletion of *yciM* is tolerated in E. coli lacking other proteins that attach the OM to the peptidoglycan, including Pal and OmpA (13). Furthermore, production of OM vesicles increases in E. coli with moderately increased levels of LPS (44), suggesting that OM vesiculation may be an adaptive response to increased LPS biosynthesis. As such, we favor the hypothesis that deletion of *lpp* lowers LPS levels by increasing OM vesiculation.

It has been proposed that transport of LPS from the IM to the OM is inhibited once a critical level of LPS within the OM is reached (45). Accordingly, it is likely that the excess LPS produced in the Δ*yciM* mutant accumulates in the IM once transport to the OM has stopped. Indeed, E. coli lacking FtsH has been shown to accumulate membranous material within the periplasm, a large portion of which is likely LPS (12). In the Δ*lpp* mutant, it is possible that transport is not inhibited because excess LPS is lost in OM vesicles. As a result, LPS accumulation within the IM would be minimized. For these reasons, we propose that it is accumulated LPS within the IM of the Δ*yciM* mutant that causes lethality and that deletion of *lpp* reduces lethality by increasing transport of LPS to the OM.

**FIG 6:**
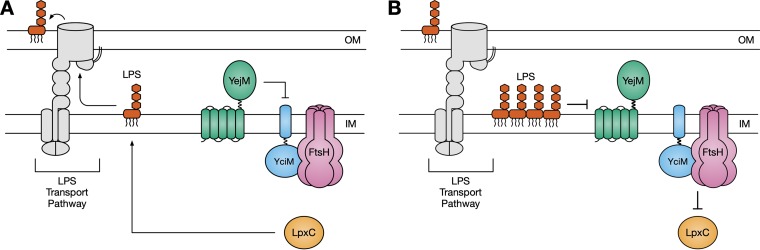
YejM alters activity of the YciM/FtsH protease complex to prevent lipopolysaccharide (LPS) accumulation in the inner membrane (IM). (A) Under normal conditions, YejM inhibits activity of the YciM/FtsH protease complex. LpxC is stabilized, which promotes LPS biosynthesis. LPS is transported to the outer membrane (OM) by the LPS transport pathway. (B) Accumulation of LPS in the outer leaflet of the IM inhibits activity of YejM. Activity of the YciM/FtsH protease complex is derepressed, leading to increased degradation of LpxC. Lower levels of LpxC reduce biosynthesis of LPS.

It stands to reason that IM LPS may be sensed by the cell, which would respond by decreasing LPS synthesis. In support of this hypothesis, it has been shown that depletion of proteins involved in LPS transport to the OM increases expression of FtsH and lowers levels of LPS (29, 46). We propose that YejM is at the center of this regulatory pathway. Under normal conditions, YejM acts to inhibit activity of YciM, which stabilizes LpxC, allowing for lipid A biosynthesis to occur (Fig. 6A). However, if LPS biosynthesis begins to outpace transport to the OM, LPS accumulates within the IM. Under these conditions, inhibition of YciM by YejM is relieved, which restores homeostasis by reducing lipid A biosynthesis (Fig. 6B). How YejM senses this event remains to be determined. Given that YejM can bind lipids (22, 23), it is possible that changes in the lipid environment of the IM upon accumulation of LPS regulate activity of YejM. This regulation would happen without the need for additional protein synthesis, allowing the cell to rapidly sense and respond to LPS that has accumulated within the IM.

Our study demonstrates that the levels of LpxC must be tightly regulated. A partial decrease in LpxC leads to defects in OM integrity and complete destruction is lethal. However, an unrestrained increase in LpxC is also lethal due to aberrant accumulation of LPS within the IM. The overall importance of this proteolytic regulatory circuit for LpxC is evidenced by the fact that three essential IM proteins, YejM, YciM, and FtsH, have been assigned to carefully balance LPS synthesis in order to maintain the OM barrier during cell growth even in rapidly changing environments.

## MATERIALS AND METHODS

### Bacterial growth conditions.

Unless otherwise stated, bacteria were cultured in Lennox broth (LB) or LB agar at 30°C. LB was supplemented with ampicillin (Amp) (125 μg ml^−1^), chloramphenicol (Cam) (20 μg ml^−1^), kanamycin (Kan) (25 μg ml^−1^), tetracycline (Tet) (10 μg ml^−1^), l-arabinose (0.2% [wt/vol] or 0.5% [wt/vol]), d-fucose (0.05% [wt/vol]), isopropyl-β-d-thiogalactopyranoside (IPTG, 2.5 μM), sodium dodecyl sulfate (SDS, 0.5% [wt/vol]), and/or EDTA (0.5 mM), as necessary.

### Strain construction.

All bacterial strains and plasmids used in this study are listed in Table S1 in the supplemental material, and all oligonucleotides are listed in Table S2. Unless otherwise stated, chromosomal mutations were introduced into E. coli strains MG1655 and JCM158 using generalized transduction (47). Alleles of *yciM* and *lpxC* were moved into E. coli JCM158 using the genetically linked markers *ycjM*::Tn*10* and *leuB*::Tn*10*, respectively, with the exception of the Δ*yciM*::*kan* null allele, which was selected for directly. To remove the *leuB*::Tn*10* marker from E. coli expressing the *lpxC*101 allele, cells were transduced with P1vir raised in wild-type E. coli and selected for on M63 minimal medium agar supplemented with 0.4% glucose, 1 mM MgSO_4_, and 125 μg ml^−1^ thiamine. Point mutations were confirmed by DNA sequencing, and null mutations were confirmed by PCR.

10.1128/mBio.00598-20.1 TABLE S1 Bacterial strains and plasmids used in this study. Download Table S1, DOCX file, 0.03 MB. Copyright © 2020 Guest et al. 2020 Guest et al. This content is distributed under the terms of the Creative Commons Attribution 4.0 International license.

10.1128/mBio.00598-20.2 TABLE S2 Oligonucleotides used in this study. Download Table S2, DOCX file, 0.02 MB. Copyright © 2020 Guest et al. 2020 Guest et al. This content is distributed under the terms of the Creative Commons Attribution 4.0 International license.

Null alleles of *yciM* and *lpp* were obtained from the Keio library (48). The kanamycin resistance cassette in the Δ*lpp*::*kan* Keio allele was removed by flippase/flippase recognition target (FLP/FRT) site-specific recombination generally as described in reference 49. pCP20, which expresses FLP from a temperature-sensitive promoter, was transformed into E. coli containing the Keio allele. Transformants were recovered in LB at 30°C for 1 h and selected for on LB agar supplemented with chloramphenicol at 30°C. The following day, single colonies were grown in LB at 37°C for 6 h to induce FLP expression and to prevent pCP20 from replicating. Serial dilutions were plated on LB medium and grown overnight at 37°C. To confirm that the bacteria had lost pCP20, colonies were screened for chloramphenicol sensitivity. The kanamycin resistance cassette in the Δ*yciM*::*kan* Keio allele was removed by FLP/FRT recombination as described in reference 50 with all steps performed at 30°C or room temperature. Loss of the kanamycin resistance cassette was confirmed by screening for kanamycin sensitivity and PCR. The scar region following FLP/FRT recombination contains a single FRT site (51).

Both the *yejM*569::*cam* and Δ*yejM*::*kan* alleles were constructed by λ Red recombination as previously described (52). To generate the *yejM*569::*cam* allele, the chloramphenicol resistance cassette and FRT sites were amplified from the plasmid pKD3 (51) using primers yejMFrecomb and yejMRrecomb. The *yejM*569::*cam* DNA was purified and transformed into strain DY378, which encodes the λ Red recombination system from a temperature-sensitive promoter. Recombinants were selected for on LB containing chloramphenicol, and the presence of the *yejM*569::*cam* allele was confirmed by PCR. To generate the Δ*yejM*::*kan* allele, the kanamycin resistance cassette and flanking FRT sites were amplified from the Keio library (48) using primers yejMKan.Fwd and yejMKan.Rev. Purified Δ*yejM*::*kan* DNA was transformed into strain RLG429, a derivative of DY378 that expresses a wild-type copy of *yejM* from an IPTG-inducible promoter on plasmid pCA-*yejM*. Recombinants were selected for on LB agar supplemented with kanamycin, chloramphenicol, and IPTG. The presence of the Δ*yejM*::*kan* allele was confirmed by PCR.

### Plasmid construction.

To construct the plasmid pBAD18-*yejM*, wild-type *yejM* was amplified from the chromosome of strain JCM158 using the oligonucleotides yejMKpnI.Fwd and yejMHindIII.Rev. Both the purified PCR product and pBAD18 were digested with KpnI (NEB) and HindIII (NEB) according to the manufacturer’s recommendations. The digested *yejM* DNA was then ligated into the digested pBAD18 vector using T4 DNA ligase (NEB) in the supplied buffer at room temperature for 1 h. Ligated plasmid was transformed into Mach1 chemically competent E. coli (Invitrogen) according to the manufacturer’s protocol. Transformants were selected for on LB agar supplemented with ampicillin. PCR and DNA sequencing were used to confirm successful cloning of *yejM* into pBAD18.

pBAD18-*lpp*_+14_ was constructed using Q5 site-directed mutagenesis (NEB). pBAD18-*lpp* was amplified with the oligonucleotides lpp14.Fwd and lpp14.Rev. The resulting PCR product was digested with DpnI overnight at 37°C in order to remove the template DNA. The following day, the 5′ ends of the linear pBAD18-lpp+14 DNA were phosphorylated using T4 polynucleotide kinase (NEB), and the DNA was circularized using T4 DNA ligase (NEB). Ligated plasmid DNA was transformed into Mach1 chemically competent E. coli (Invitrogen) according to the manufacturer’s protocol, and transformants were selected for on LB agar supplemented with ampicillin at 37°C. The resulting *lpp*_+14_ allele contains a TLSAKVEQLSNDVN insertion between Lpp^D42^ and Lpp^Q43^, which extends the length of Lpp by 14 amino acids (36). The pBAD18-*lpp*_+21_ plasmid was constructed using a similar procedure; however, pBAD18-*lpp* was instead amplified with the primers lpp21.Fwd and lpp21.Rev. Lpp^+21^ contains a TLSAKVEQLSNDVNAMRSDVD insertion between residues D42 and Q43 and extends the protein by 21 amino acids (36). Mutagenesis of pBAD18-*lpp*_+14_ and pBAD18-*lpp*_+21_ was confirmed by DNA sequencing.

### Isolation and identification of suppressor mutations.

Overnight cultures of E. coli expressing *yejM*569 were plated on LB agar at 42°C or LB agar containing 0.5% SDS and 0.5 mM ETDA at 30°C and incubated until colonies formed. Suppressor mutations in LpxC were identified by whole-genome sequencing. Genomic DNA of the parent strain and the suppressors was isolated using the DNeasy blood and tissue kit (Qiagen) following the manufacturer’s protocol for isolation of DNA from Gram-negative bacteria. An Illumina (CA) sequencing library of the DNA was prepared using the Nextera DNA library prep kit and was sequenced using an Illumina HiSeq 2500 sequencer. Whole-genome sequencing and analysis were performed by the Princeton University Lewis-Sigler Institute Genomics Core Facility. Mutations in *yciM* were identified by Sanger sequencing (Genewiz) using the primers yciM_F and yciM_R.

### Efficiency of plating.

Bacterial cultures grown overnight were standardized by optical density at 600 nm (OD_600_) and then serially diluted by a factor of 10 in a 96-well plate. Bacteria were transferred to the indicated agar medium using a 96-well plate replica plater and grown overnight at 30°C unless otherwise stated.

### Immunoblot analysis.

For experiments with E. coli expressing *yejM*569, bacteria were grown to an OD_600_ of 0.4 to 0.6 in 5 ml LB at 30°C in a roller drum. Three milliliters of culture was collected, and bacteria were pelleted by centrifugation at 21,130 × *g* for 1 min at room temperature. The supernatant was removed, and bacteria were flash frozen in liquid nitrogen. Frozen pellets were resuspended in lysis buffer (25 mM Tris [pH 6.8] with 1% SDS) and boiled for 5 min. The protein concentration of each sample was measured using the Pierce BCA protein assay kit (ThermoFisher). Samples were standardized by protein concentration, diluted twofold in sample buffer (125 mM Tris [pH 6.8], 2.5% glycerol, 3% SDS, 0.5 mg/ml bromophenol blue, 4% β-mercaptoethanol), and electrophoresed on a 10% SDS-polyacrylamide gel. To look at LPS levels in the Δ*yciM* mutants, bacteria were grown overnight in 200 μl LB containing Amp and 0.5% l-arabinose in a 96-well plate at 30°C as described below for growth curves. Bacteria were concentrated to an OD_600_ of 1.25 in sample buffer and lysed by boiling for 5 min. Samples were electrophoresed on a 10% or 12% SDS-polyacrylamide gel. LPS and proteins were transferred to a nitrocellulose membrane using the Trans-Blot Turbo Transfer System (Bio-Rad). Primary antibodies detecting LPS core (Hycult Biotech), LpxC, OmpC/F/A, RecA, and RpoA were used at a 1:5,000, 1:10,000, 1:30,000, 1:10,000, and 1:50,000 dilution, respectively. Goat anti-mouse horseradish peroxidase (HRP) conjugate (Bio-Rad) and goat anti-rabbit peroxidase (Sigma-Aldrich) secondary antibodies were each used at a 1:10,000 dilution.

### Growth curve.

Overnight cultures of bacteria grown in 5 ml LB supplemented with Amp and d-fucose were diluted to an OD_600_ of approximately 0.02 in 2 ml LB supplemented with Amp and 0.5% l-arabinose. Two hundred microliters of each culture was transferred to each well on a 96-well plate, which was then covered with a Breathe-Easy gas-permeable membrane (Sigma-Aldrich). Bacteria were grown overnight in the Synergy H1 microplate reader (BioTek) at 30°C with shaking. OD_600_ was measured every 15 min for 12 h.

### Fractionation of OM vesicles.

OM vesicles were collected as described in reference 29. Bacteria were grown for 16 h in 5 ml LB at 30°C. Bacteria equivalent to an OD_600_ of 1 were pelleted by centrifugation at 21,130 × *g* for 1 min at room temperature and resuspended in 100 μl of sample buffer and lysed by boiling for 5 min. Bacteria in the remainder of the culture were pelleted by centrifugation at approximately 3,500 rpm for 10 min at 4°C. The supernatant was then filtered through a 0.2-μm filter. Supernatant equivalent to an OD_600_ of 3 was collected, and the volume was equalized between samples with fresh LB. The supernatants were then filtered through an Amicon Ultra-15 centrifugal filter unit (Millipore) with a molecular weight cutoff of 100 kDa. Samples were resuspended in 50 μl and boiled for 5 min. Proteins were separated by electrophoresis on a 10% SDS-polyacrylamide gel and detected via immunoblot analysis.
