# Supplementary material for: YejM Modulates Activity of the YciM/FtsH Protease Complex To Prevent Lethal Accumulation of Lipopolysaccharide
Source: mBio. 2020 Apr 14;11(2):e00598-20. doi: 10.1128/mBio.00598-20 (PMC7157816; doi:10.1128/mBio.00598-20)
Supplement: TABLE S1 [file mBio.00598-20-st001.docx]

| Table S1. Bacterial strains and plasmids used in this study. | | |
| --- | --- | --- |
| Strain or plasmid | Description | Source or reference |
| *Bacterial strains* | | |
| MC4100 | F-*araD139* (*argF-lac*)*U169* *rpsL150 relA1 flb5301 deoC1 ptsF25 thi* | (1) |
| JCM158 | MC4100 ara^R^ | (2) |
| MG1655 | K-12 F^-^ λ^-^ *rph-1* | (3) |
| DY378 | W3110 λ*c*I*857* Δ*(cro‐bioA)* | (4) |
| MW06 | MG1655 *yejM*569::*kan* | (5) |
| MW13 | DY378 *yejM*569::*cam* | This study |
| MW15 | MG1655 *yejM*569::*cam* | This study |
| JW1272 | BW25113 *lpxC*1272 Δ*yciM*::*kan* | (6) |
| JW1667 | BW25113 Δ*lpp*::*kan* | (6) |
| RLG256 | BW25113 *lpxC*1272 Δ*yciM*::*kan* *ycjM*::Tn10 | This study |
| RLG429 | DY378 Δ*yejM*::*kan* (pCA-*yejM*) | This study |
| RLG431 | JCM158 *ycjM*::Tn10 | This study |
| RLG433 | JCM158 *leuB::*Tn10 | This study |
| RLG438 | JCM158 *lpxC*101 | This study |
| RLG440 | JCM158 (pBAD18-*yejM*) | This study |
| RLG441 | JCM158 *ycjM*::Tn10 (pBAD18-*yejM*) | This study |
| RLG442 | JCM158 *yciM_V43G_ ycjM*::Tn10 (pBAD18-*yejM*) | This study |
| RLG445 | JCM158 Δ*lpp*::FRT | This study |
| RLG455 | JCM158 **Δ***yejM*::*kan* (pBAD18-*yejM*) | This study |
| RLG465 | JCM158 *yejM*569::*cam* | This study |
| RLG467 | JCM158 *ycjM*::Tn10 *yejM*569::*cam* | This study |
| RLG469 | JCM158 *yejM*569::*cam* (pBAD18-*yejM*) | This study |
| RLG470 | JCM158 *ycjM*::Tn10 *yejM*569::*cam* (pBAD18-*yejM*) | This study |
| RLG471 | JCM158 *yciM*_V43G_ *ycjM*::Tn10 *yejM569*::*cam* (pBAD18-*yejM*) | This study |
| RLG472 | JCM158 *lpxC*101 (pBAD18-*yejM*) | This study |
| RLG480 | JCM158 *lpxC*101 *yejM*569::*cam* (pBAD18-*yejM*) | This study |
| RLG507 | JCM158 *yciM*_A143E_ *ycjM*::Tn10 *yejM*569::*cam* | This study |
| RLG509 | JCM158 *yciM*_A376P_ *ycjM*::Tn10 *yejM*569::*cam* | This study |
| RLG511 | JCM158 *yciM*_*390Q_ *ycjM*::Tn10 *yejM*569::*cam* | This study |
| RLG547 | JCM158 *leuB::*Tn10 *yejM*569::*cam* | This study |
| RLG548 | JCM158 *leuB::*Tn10 *lpxC*_R230L_ *yejM*569::*cam* | This study |
| RLG550 | JCM158 *leuB::*Tn10 *lpxC*_*306_*_fs_*_A_ *yejM*569::*cam* | This study |
| RLG555 | JCM158 *lpxC*101 Δ*yciM*::FRT (pBAD18-*yejM*) | This study |
| RLG557 | JCM158 Δ*yciM*::FRT Δ*lpp*::FRT (pBAD18-*yejM*) | This study |
| RLG558 | JCM158 *lpxC*101 Δ*yciM*::FRT *yejM*569::*cam* (pBAD18-*yejM*) | This study |
| RLG560 | JCM158 *lpxC*101 **Δ***yejM*::*kan* (pBAD18-*yejM*) | This study |
| RLG562 | JCM158 *lpxC*101 Δ*yciM*::FRT **Δ***yejM*::*kan* (pBAD18-*yejM*) | This study |
| RLG564 | JCM158 Δ*yciM*::FRT **Δ***lpp*::FRT (pBAD18) | This study |
| RLG565 | JCM158 Δ*yciM*::FRT **Δ***lpp*::FRT (pBAD18-*lpp*) | This study |
| RLG566 | JCM158 Δ*yciM*::FRT **Δ***lpp*::FRT (pBAD18-*lpp*_+14_) | This study |
| RLG568 | JCM158 Δ*yciM*::FRT **Δ***lpp*::FRT (pBAD18-*lpp*_ΔK58_) | This study |
| RLG569 | JCM158 **Δ***lpp*::FRT (pBAD18-*yejM*) | This study |
| RLG573 | JCM158 Δ*yciM*::FRT **Δ***lpp*::FRT **Δ***yejM*::*kan* (pBAD18-*yejM*) | This study |
| RLG574 | JCM158 **Δ***lpp*::FRT (pBAD18) | This study |
| RLG575 | JCM158 **Δ***lpp*::FRT (pBAD18-*lpp*) | This study |
| RLG576 | JCM158 **Δ***lpp*::FRT (pBAD18-*lpp*_+14_) | This study |
| RLG577 | JCM158 **Δ***lpp*::FRT (pBAD18-*lpp*_+21_) | This study |
| RLG578 | JCM158 **Δ***lpp*::FRT (pBAD18-*lpp*_ΔK58_) | This study |
| RLG587 | JCM158 **Δ***lpp*::FRT (pBAD18-*yejM*) | This study |
| RLG588 | JCM158 **Δ***lpp*::FRT Δ*yejM*::FRT (pBAD18-*yejM*) | This study |
|  |  |  |
| *Plasmids* | | |
| pBAD18 | Vector containing the P*_araB_* arabinose-inducible promoter | (7) |
| pBAD18-*yejM* | Arabinose-inducible *yejM* overexpression vector | This study |
| pBAD18-*lpp* | Arabinose-inducible *lpp* overexpression vector | (8) |
| pBAD18-*lpp*_+14_ | Arabinose-inducible *lpp*_+14_ overexpression vector | This study |
| pBAD18-*lpp*_+21_ | Arabinose-inducible *lpp*_+21_ overexpression vector | This study |
| pBAD18-*lpp*_ΔK58_ | Arabinose-inducible *lpp*_ΔK58_ overexpression vector | (8) |
| pCA-*yejM* | IPTG-inducible *yejM* overexpression vector from the ASKA library | (9) |
| pFLP2 | Temperature sensitive flippase expression vector | (10) |
| pCP20 | Temperature sensitive flippase expression vector | (11) |
| pKD3 | Plasmid containing a chloramphenicol resistance cassette flanked by FRT sites | (12) |

**References**

1. **Casadaban MJ**. 1976. Transposition and fusion of the lac genes to selected promoters in Escherichia coli using bacteriophage lambda and Mu. J Mol Biol **104**:541–555.

2. **Malinverni JC**, **Werner J**, **Kim S**, **Sklar JG**, **Kahne D**, **Misra R**, **Silhavy TJ**. 2006. YfiO stabilizes the YaeT complex and is essential for outer membrane protein assembly in Escherichia coli. Molecular Microbiology **61**:151–164.

3. **Guyer MS**, **Reed RR**, **Steitz JA**, **Low KB**. 1981. Identification of a sex-factor-affinity site in E. coli as gamma delta. Cold Spring Harb Symp Quant Biol **45 Pt 1**:135–140.

4. **Yu D**, **Ellis HM**, **Lee EC**, **Jenkins NA**, **Copeland NG**, **Court DL**. 2000. An efficient recombination system for chromosome engineering in Escherichia coli. Proc Natl Acad Sci USA **97**:5978–5983.

5. **De Lay NR**, **Cronan JE**. 2008. Genetic interaction between the Escherichia coli AcpT phosphopantetheinyl transferase and the YejM inner membrane protein. Genetics **178**:1327–1337.

6. **Baba T**, **Ara T**, **Hasegawa M**, **Takai Y**, **Okumura Y**, **Baba M**, **Datsenko KA**, **Tomita M**, **Wanner BL**, **Mori H**. 2006. Construction of Escherichia coli K-12 in-frame, single-gene knockout mutants: the Keio collection. Mol Syst Biol **2**:2006.0008.

7. **Guzman LM**, **Belin D**, **Carson MJ**, **Beckwith J**. 1995. Tight regulation, modulation, and high-level expression by vectors containing the arabinose PBAD promoter. J Bacteriol **177**:4121–4130.

8. **Cowles CE**, **Li Y**, **Semmelhack MF**, **Cristea IM**, **Silhavy TJ**. 2011. The free and bound forms of Lpp occupy distinct subcellular locations in Escherichia coli. Molecular Microbiology **79**:1168–1181.

9. **Kitagawa M**, **Ara T**, **Arifuzzaman M**, **Ioka-Nakamichi T**, **Inamoto E**, **Toyonaga H**, **Mori H**. 2005. Complete set of ORF clones of Escherichia coli ASKA library (a complete set of E. coli K-12 ORF archive): unique resources for biological research. DNA Res **12**:291–299.

10. **Hoang TT**, **Karkhoff-Schweizer RR**, **Kutchma AJ**, **Schweizer HP**. 1998. A broad-host-range Flp-FRT recombination system for site-specific excision of chromosomally-located DNA sequences: application for isolation of unmarked Pseudomonas aeruginosa mutants. Gene **212**:77–86.

11. **Cherepanov PP**, **Wackernagel W**. 1995. Gene disruption in Escherichia coli: TcR and KmR cassettes with the option of Flp-catalyzed excision of the antibiotic-resistance determinant. Gene **158**:9–14.

12. **Datsenko KA**, **Wanner BL**. 2000. One-step inactivation of chromosomal genes in Escherichia coli K-12 using PCR products. Proc Natl Acad Sci USA **97**:6640–6645.
