# Supplementary material for: YejM Modulates Activity of the YciM/FtsH Protease Complex To Prevent Lethal Accumulation of Lipopolysaccharide
Source: mBio. 2020 Apr 14;11(2):e00598-20. doi: 10.1128/mBio.00598-20 (PMC7157816; doi:10.1128/mBio.00598-20)
Supplement: TABLE S2 [file mBio.00598-20-st002.docx]

| Table S2: Oligonucleotides used in this study. | |
| --- | --- |
| Primer name | DNA sequence (5’-3’)* |
| lpp14.Fwd | caactgtccaatgatgtaaatCAGCTGAGCAACGACGTG |
| lpp14.Rev | ctccactttggcactcagagtGTCAACTTTAGCGTTCAGAGTC |
| lpp21.Fwd | caatgatgtaaatgcgatgcgttcagacgttgatCAGCTGAGCAACGACGTG |
| lpp21.Rev | gacagttgctccactttggcactcagagtGTCAACTTTAGCGTTCAGAGTC |
| yciM_F | CTGGAAAACCAGCTTTCACC |
| yciM_R | TAAACGGCGAGCATTCTACC |
| yejM569Frecomb | TGGCCGCATTCTTATTTATCGCCTTTATCGCCTCGCATGTGGTGTATATCTGATAGGTGTAGGCTGGAGCTGCTTC |
| yejM569Rrecomb | TTTCCACACCGATTGCAAGTAAGATATTTCGCTAACTGATTTATAATTAAATGGGAATTAGCCATGGTCC |
| yejMKpnI.Fwd | TTTTGGTACCTATCAACGAAGACAAAGCGC |
| yejMHindIII.Rev | TTTTAAGCTTGACTCGTGGCTGCTAATACTAC |
| yejMKan.Fwd | CTCTATCAACGAAGACAAAGCGCACTAAGGGAAACAGATAACAGGTTatgATTCCGGGGATCCGTCGACC |
| yejMKan.Rev | AGATATTTCGCTAACTGATTTATAATTAAtcaGTTAGCGATAAAACGCTTTGTAGGCTGGAGCTGCTTCG |
| *Underlined sequences denote restriction enzyme cut sites (KpnI: GGTACC, HindIII: AAGCTT). | |
